# Supplementary material for: Revealing the assembly of filamentous proteins with scanning transmission electron microscopy
Source: PLoS One. 2019 Dec 20;14(12):e0226277. doi: 10.1371/journal.pone.0226277 (PMC6924676; doi:10.1371/journal.pone.0226277)
Supplement: S1 Fig — (PDF) [file pone.0226277.s001.pdf]

Revealing the assembly of filamentous proteins with scanning transmission electron microscopy

Cristina Martinez-Torres[1,2], Federica Burla[1], Celine Alkemade[1,2], Gijsje H. Koenderink[1,2]*

[1]Department of Living Matter, AMOLF, Amsterdam, the Netherlands

[2]Department of Bionanoscience, Kavli Institute of Nanoscience Delft, Faculty of Applied Sciences, Delft University of Technology, Delft, The Netherlands

* E-mail: g.h.koenderink@tudelft.nl

Supporting Figure 1

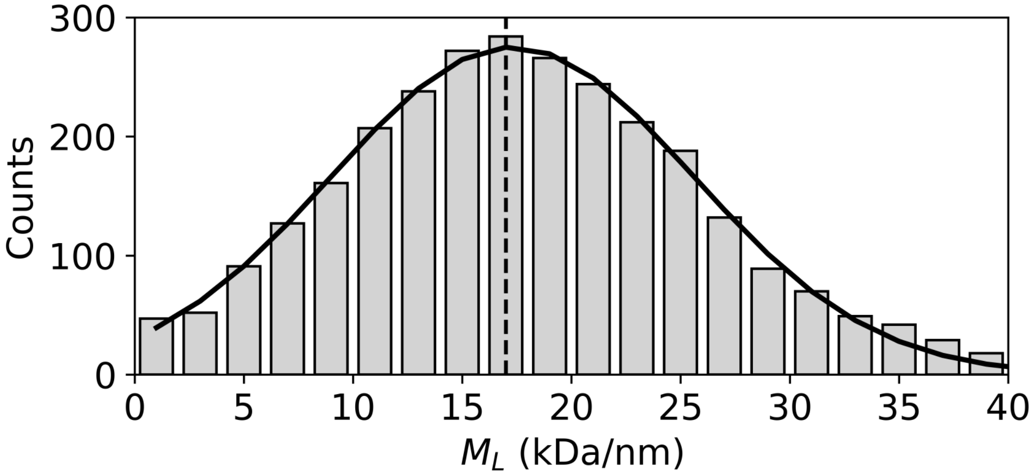

S1 Fig. Distribution of mass per length values for Fd-bacteriophage filaments, used as an internal calibration standard. The solid black line shows a Gaussian curve fit with an average centered around $<M_L> = 17.3 \pm 8.2$ kDa/nm, and the dashed line shows the M_L value (17 kDa/nm) known from the virus structure.[1]

References Supporting Figure 1

1. Zimmerman, K.; Hagedorn, H.; Heuck, C.C.; Hinrichsen, M.; Ludwig, H. The Ionic Properties of the Filamentous Bacteriophages Pf1 and Fd*. J. Biol. Chem. 1986, 261, 1653-1655.
