# Supplementary material for: Revealing the assembly of filamentous proteins with scanning transmission electron microscopy
Source: PLoS One. 2019 Dec 20;14(12):e0226277. doi: 10.1371/journal.pone.0226277 (PMC6924676; doi:10.1371/journal.pone.0226277)
Supplement: S2 Fig — (PDF) [file pone.0226277.s002.pdf]

Revealing the assembly of filamentous proteins with scanning transmission electron microscopy

Cristina Martinez-Torres[1,2], Federica Burla[1], Celine Alkemade[1,2], Gijsje H. Koenderink[1,2]*

[1]Department of Living Matter, AMOLF, Amsterdam, the Netherlands

[2]Department of Bionanoscience, Kavli Institute of Nanoscience Delft, Faculty of Applied Sciences, Delft University of Technology, Delft, The Netherlands

* E-mail: g.h.koenderink@tudelft.nl

Supporting Figure 2

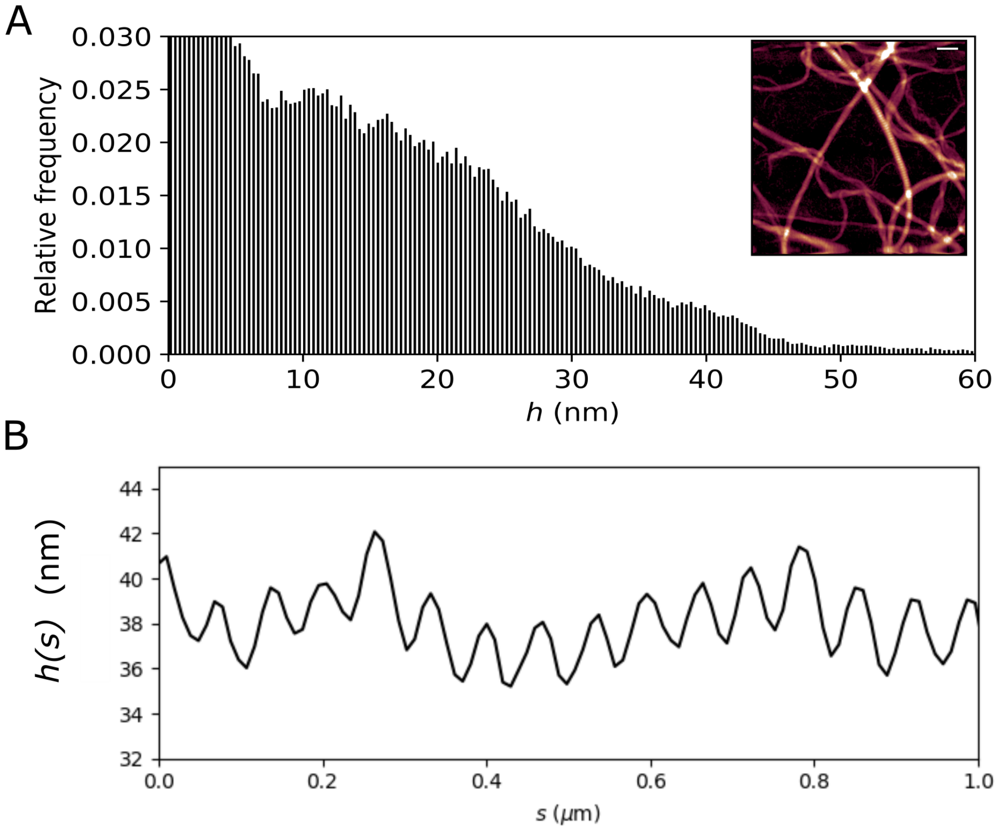

S2 Fig. Atomic force microscopy of collagen fibrils. (A) Distribution of height *(h)* values for the AFM image of collagen fibrils shown in the inset and in Fig. 3B. There is a large majority of thin fibrils (up to 45 nm) and a few thicker ones (up to 60 nm). (B) Representative height profile for a fibril showing the characteristic D-banded pattern, where the height oscillations correspond to the D-periodicity of gap and overlap regions with an average spacing $\langle D \rangle = 65.4 \pm 5.4$ nm.
