# Supplementary material for: Revealing the assembly of filamentous proteins with scanning transmission electron microscopy
Source: PLoS One. 2019 Dec 20;14(12):e0226277. doi: 10.1371/journal.pone.0226277 (PMC6924676; doi:10.1371/journal.pone.0226277)
Supplement: S3 Fig — (PDF) [file pone.0226277.s003.pdf]

Revealing the assembly of filamentous proteins with scanning transmission electron microscopy

Cristina Martinez-Torres[1,2], Federica Burla[1], Celine Alkemade[1,2], Gijsje H. Koenderink[1,2]*

[1]Department of Living Matter, AMOLF, Amsterdam, the Netherlands

[2]Department of Bionanoscience, Kavli Institute of Nanoscience Delft, Faculty of Applied Sciences, Delft University of Technology, Delft, The Netherlands

* E-mail: g.h.koenderink@tudelft.nl

Supporting Figure 3

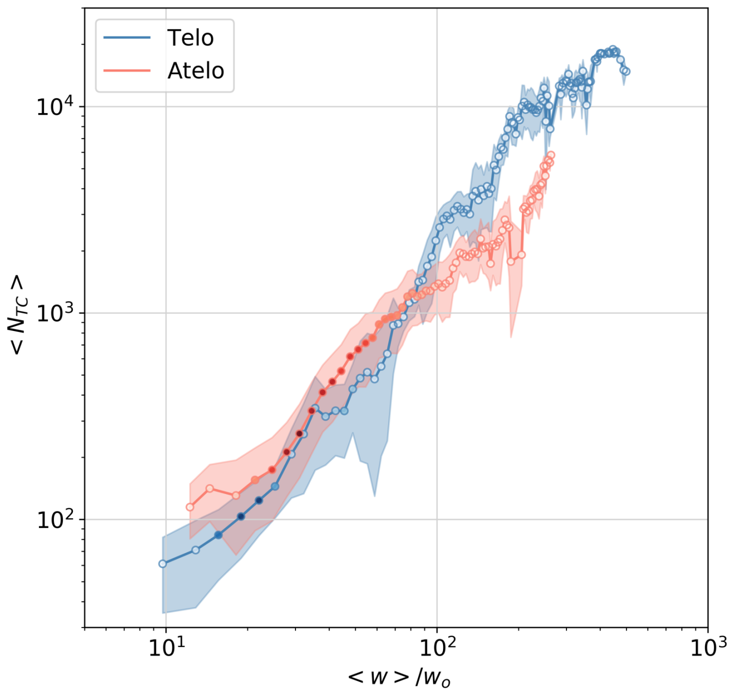

S3 Fig. **Collagen fibril packing regimes.** Average number of tropocollagen molecules per cross section, N_{TC} as a function of the normalized fibril width w/w_o, with w_o = 1.5 nm, m_o = 1.15 kDa/nm for atelo- and m_o = 1.17 kDa/nm for telo- collagen. The error bars represent the standard deviation. n = 15695 (telocollagen) and n = 41730 (atelocollagen) segments. Same data as in Fig. 4B in log-log representation and normalized.
