# Supplementary material for: Revealing the assembly of filamentous proteins with scanning transmission electron microscopy
Source: PLoS One. 2019 Dec 20;14(12):e0226277. doi: 10.1371/journal.pone.0226277 (PMC6924676; doi:10.1371/journal.pone.0226277)

Revealing the assembly of filamentous proteins with scanning transmission electron microscopy

Cristina Martinez-Torres[1,2], Federica Burla[1], Celine Alkemade[1,2], Gijsje H. Koenderink[1,2]*

[1]Department of Living Matter, AMOLF, Amsterdam, the Netherlands

[2]Department of Bionanoscience, Kavli Institute of Nanoscience Delft, Faculty of Applied Sciences, Delft University of Technology, Delft, The Netherlands

* E-mail: g.h.koenderink@tudelft.nl

Supporting Figure 4

S4 Fig. Compilation of HAADF images from telocollagen (left) and atelocollagen (right) fibrils. Each column shows 5 random images taken from the dataset of each type of collagen. Note that both collagen types form polymorphic fibrils that vary in width and M_L, and in the absence or presence of D-banding.

Telocollagen | Atelocollagen

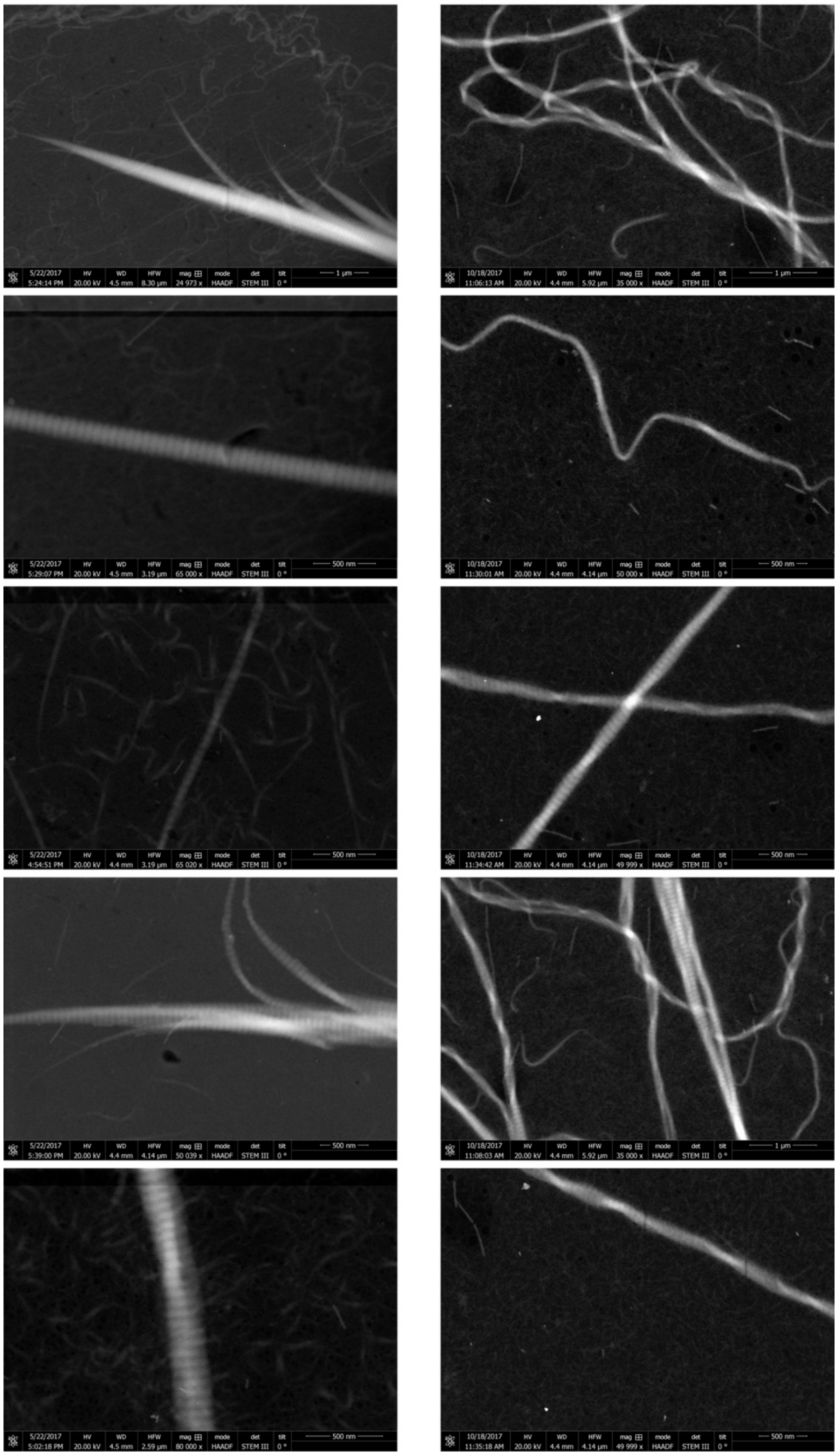

Supplement: S4 Fig — (PDF) [file pone.0226277.s004.pdf]
