# Supplementary material for: Revealing the assembly of filamentous proteins with scanning transmission electron microscopy
Source: PLoS One. 2019 Dec 20;14(12):e0226277. doi: 10.1371/journal.pone.0226277 (PMC6924676; doi:10.1371/journal.pone.0226277)
Supplement: S1 File — (PDF) [file pone.0226277.s005.pdf]

# fiAS

v0.1.0

Quick Guide

Cristina Martinez-Torres

November 4, 2019

# 1 Getting Started

## 1.1 Requirements

The program is written in python 3.5, and it needs a few packages to work properly, although most of them, if not all, should already be installed:

- tkinter
- numpy
- matplotlib
- Pillow
- scipy
- scikit-image

**NOTE: Issue with Anaconda packages**

If python has been installed using the Anaconda distribution, and the code is running on Windows, there might be a compatibility issue. To solve this, you must uninstall `matplotlib` and `numpy`, and reinstall using `pip`. In the command line, type:

```
>> conda uninstall matplotlib
>> conda uninstall numpy
>> pip install matplotlib
>> pip install numpy
```

Note that uninstalling and reinstalling packages will affect your dependencies. If this occurs, you will need to solve conflicts with dependencies individually.

## 1.2 Installation and Usage

The full code (GUI and other functions) is contained in the folder `fias`. Given the length of the code, splitting into different files allows for an easier maintenance and troubleshooting. Likewise, it is more straightforward to implement new features and modules.

Although the code can be run directly from the terminal without being installed, it is recommended to install it before running, to assure all the dependencies are fulfilled. Otherwise, the code might crash in an advanced stage of the data analysis, due to a missed package.

**fiAS installation**

To install the package, move to the directory where fiAS is located, and install using `pip`. In the example below, I have downloaded the code as a folder named `fias-master`, on the `D:` drive:

```
D:\> cd fias-master
D:\fias-master> pip install fias
```

The package is installed successfully if all the requirements are met. If there are any dependencies missing, `pip` will try to install them. Once you have sucessfully installed the package, it is ready for use.

The GUI consists of two independent main modules: `fiAS_track.py` and `fiAS_an.py`. As its name suggests, the first module is used for tracking the filaments and extracting the intensity profiles (and complementary data) that will later be used for analysis in the second module, where mass mapping can be done.

**Run fiAS**

To launch the program, you just need to tell python to run one code, depending whether you want to work on the tracking module, or the analysis module.

To run the tracking GUI, on the command line type:

```
>> python "fias\fias_track.py"
```

To run the analysis/mass mapping GUI, on the command line type:

```
>> python "fias\fias_an.py"
```

Note that the above example works only if you're already in the folder where the code is stored. Otherwise, you'll need to specify the full path where it is.

**NOTE: GUI crash with macOS**

There are some issues reported on macOS Mojave running versions of python $> 3.7$, although the issues might extend to other macOS/python versions. The issues encountered are typically the crash of the GUI and/or forced session logout of macOS. One possible solution is to work with a virtual environment using a previous python version. If conda is being used, on the terminal type:

```
>> conda create -n visual python=3.7.0
>> conda activate visual
```

Here `visual` is the name of the virtual environment created, and can be changed according to your preference.

## 2 Tracking Module

As soon as the tracking module is launched (see above), the window for the semi-automated tracking of filaments will appear (Fig. 1). Note that most of the buttons and features are disabled, as they cannot be operated at this time. In principle, at each step of interaction with the GUI, only those buttons and functions that can be performed are activated. In practice, this is not always true, so if you click on something and nothing happens, it could be that nothing is meant to happen at that particular moment (e.g., you cannot filter an image if you have not loaded it first). In the cases where it is not so clear why nothing happened

(mostly on the data analysis part), you could try and see in the terminal what's the error message.

The Tracking module has 2 windows: the main window with a menu bar, where the image will be displayed, and a float window acting as 'control panel' in this module, containing all the parameters that can be tuned and the necessary actions for an appropriate tracking.

In this (not so) quick guide, I will not go into full detail on the inner workings of the algorithm on tracking works. Instead, I'll just explain the most basic concepts and features, by analysing one example image. When possible, I'll include examples of good and bad performance using different images to illustrate.

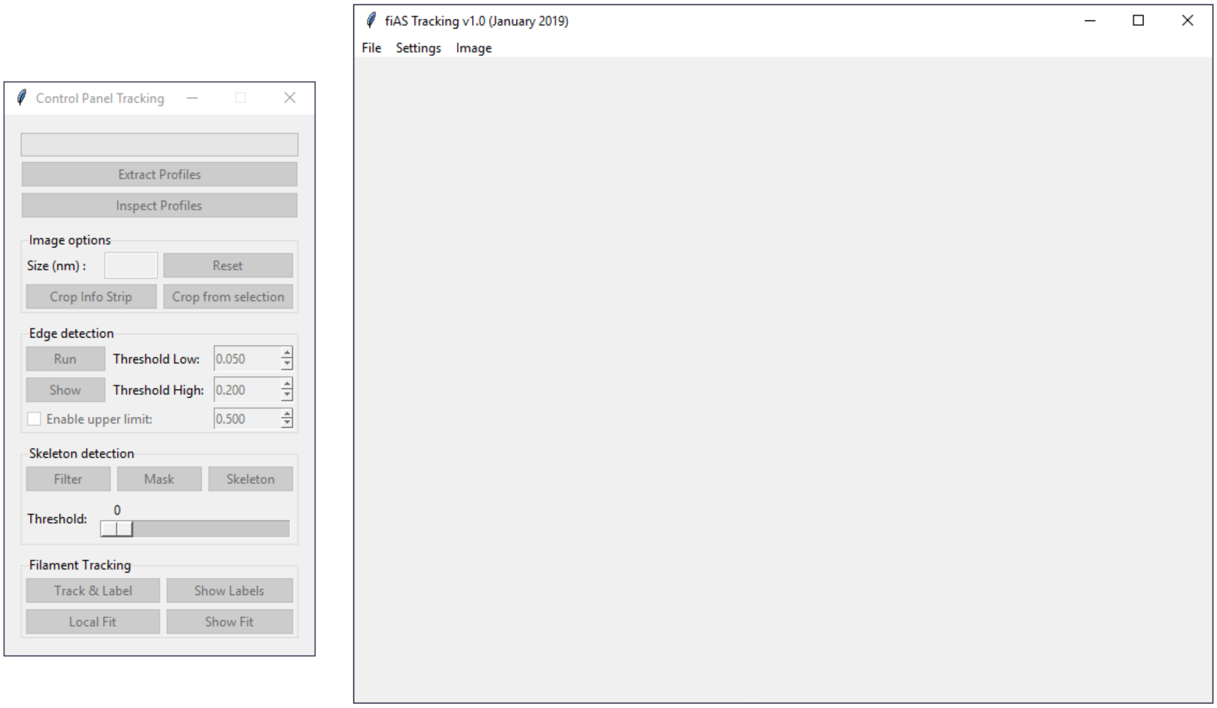


Figure 1: **Tracking Module after launch.** The control panel is a non-resizable floating window that is always present when the tracking module is being used (it can only be minimized)

## 2.1 Image load and cropping

**Quick instructions: image loading and cropping**

```
[Menu] -> File -> Open
[Control Panel] [Image Options]-> Enter Image size -> <Return>
[Control Panel] [Image Options] -> Crop Info Strip
```

First step is to load an image. The program accepts TIFF images with either the extensions: `.tif` or `.tiff`. In the menu bar, go to `File->Open`. A file dialog will appear where you can browse through the directories and select your image. As soon as the image is loaded, it will be displayed in the central panel (Fig. 2), and the information relative to the file will be updated. **Tip:** To see the image information, go to `Image-> Show Image Information`.

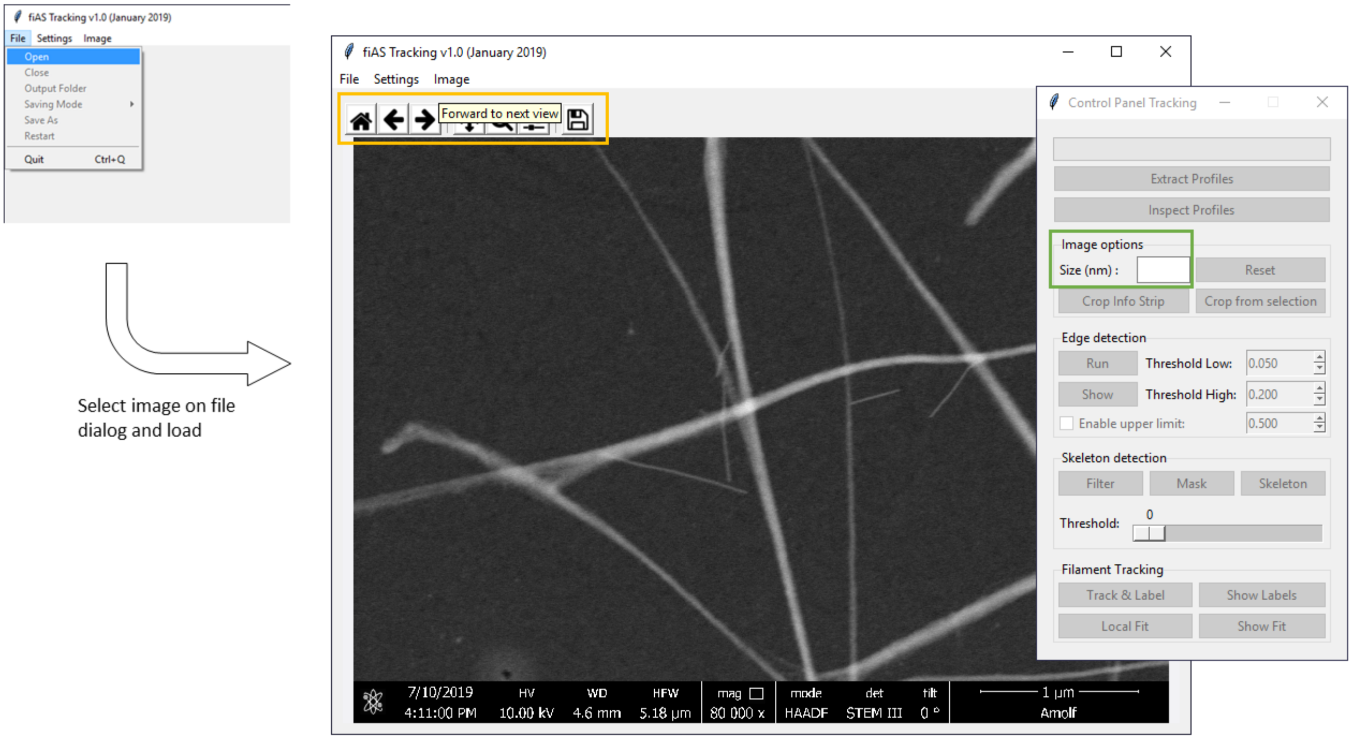


Figure 2: **Load an image in the tracking module.** On the menu bar go to `File -> Open` (shown in the upper left corner). The image loaded will be displayed on the main window. The standard toolbar from `matplotlib` (highlighted with the orange rectangle) is added so zooming, panning and saving the image are all possible. Once an image is loaded, the `Size entry` option in the control panel (highlighted with the green rectangle) will be available.

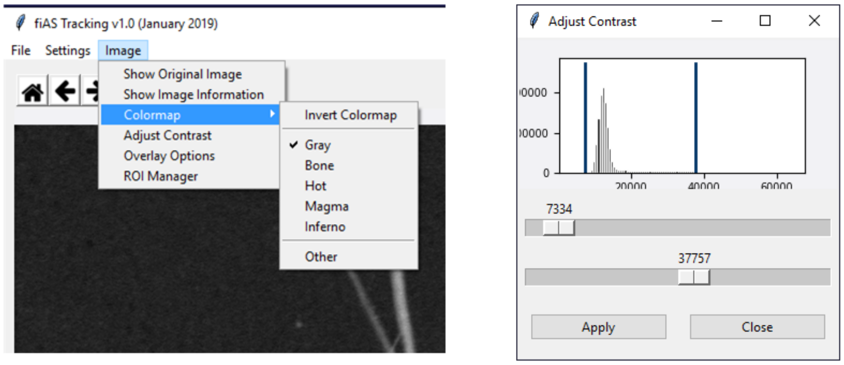


Figure 3: **Colormap and contrast options on the tracking module.** By default, the colormap of choice is in grayscale, but this can be modified, as well as the colour code (both options in the `Image` menu: `Colormap` and `Adjust Contrast`)

The only feature that will be enabled in the `Control Panel` window, is the entry for the image size in the `Image options` panel (Fig. 2). To unblock the next step, you must enter the size indicated on the STEM image bar, under `HFW` (in nm). When you hit `<Return>` on the keyboard, a few more options will activate.

It is now possible to proceed to crop the image. There are two methods to crop the image: removing only the information strip, or by cropping from a selected area (Fig. 4). The first option, `Crop Info Strip`, crops a given number of lines on the image, if the size of the information strip is known (predefined size image: 3072x2032). The second option, `Crop from selection`, requires to first select an area of the image using the display toolbar, by using the `Zoom` option. The part of the image currently shown in the display, is the part taken into account as the selection. Note that you can always go back to the original image by the `Reset` button. **Tip:** It is possible to select or delete points and tracked filaments by manually drawing ROIs on the image (`[Menu] -> Image-> ROI Manager`)

Once the image is cropped, the tracking options for both the filament edges and the skeleton, will be enabled (Fig. 4)

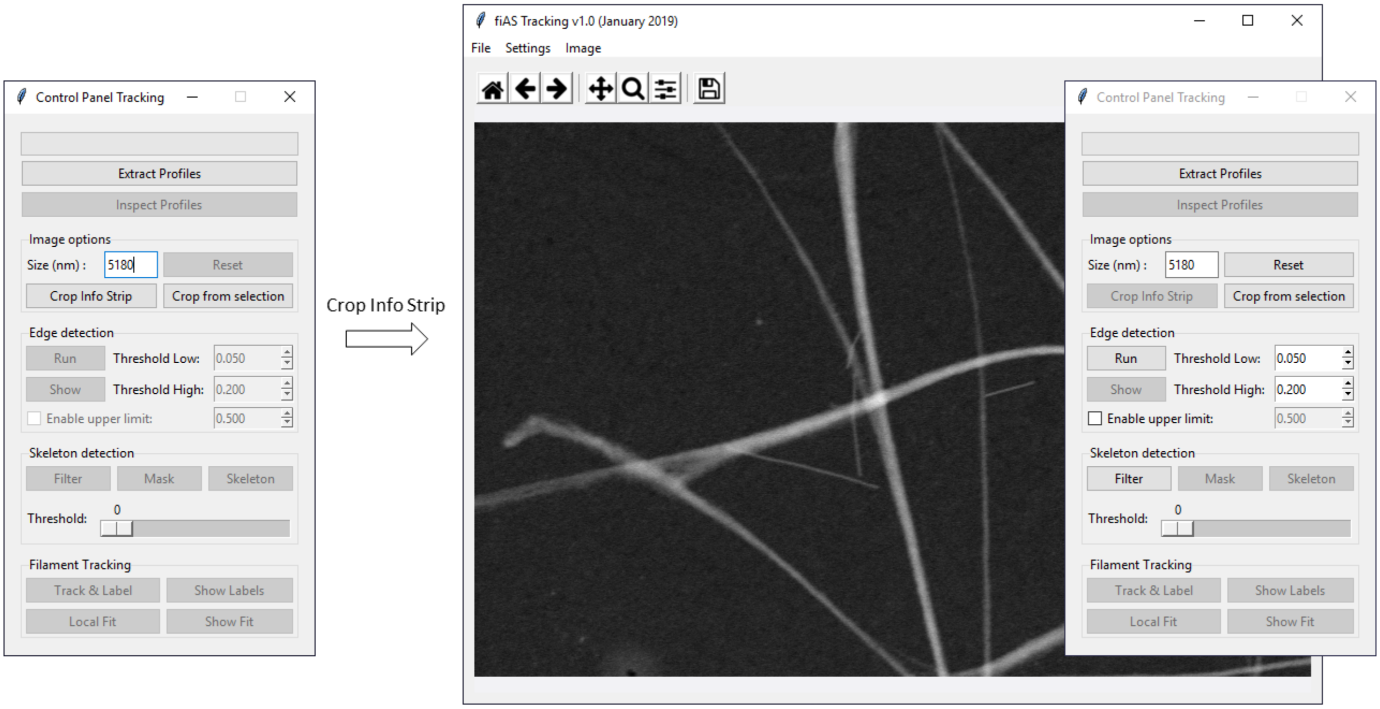


Figure 4: **Cropping the image.** After the size is known, it is possible to crop the image. Then, the options for edge detection and skeleton detection will unlock.

**Known Issue: Cropping from selected area**

In the current version, there is an issue with the edge tracking in images that have been cropped by selecting an area. Thus, it is strongly recommended not to use this option without testing it. The issue arises because the edge tracking algorithm relies on operating in Fourier space, and this is computationally delicate when dealing with odd numbers. If the size of the resulting cropped image is **NOT** an even number, it will likely result in problems with the edge tracking.

## 2.2 Edge Detection

**Quick instructions: Edge Detection**

`[Control Panel] [Edge detection] -> Run`

Check if detected edges are ok, if not, optimise threshold

`[Control Panel] [Edge detection][Theshold High] -> new value -> <Return>`
`[Control Panel] [Edge detection] -> Run`

The detection of filament edges is based on a modified canny edge detector with the wavelet transform. The idea of this method is to convolve the image with a kernel consisting of the first derivative of a gaussian, resulting in the smoothed version of the 2D gradient of the image. Then, a double hysteresis algorithm is applied to identify the true edges.

In practice, there are two main parameters to optimise: the width or scale of the gaussian envelope of the kernel, and the high threshold value. The scale of the gaussian envelope can be found in the advanced settings (`[Menu] -> Settings -> Advanced Settings`), as the value increases, the smoothing is stronger. The default value is `6`, which is the minimum value to have a well defined kernel and which works for most of the datasets, however in some cases it might be required to smooth the image further in order to avoid the detection of 'too many' edges. Note that the edge detection works by extracting the position where the gradient is maximal, therefore, if the width of the gaussian kernel is too large, the filament edges will have a relative widening or offset from the true edge (Fig. 5). It is therefore recommended to keep the value as low as possible, and to always save the settings that were used to perfom the edge detection. If the gaussian scale is known, it is then possible to correct for the offset introduced.

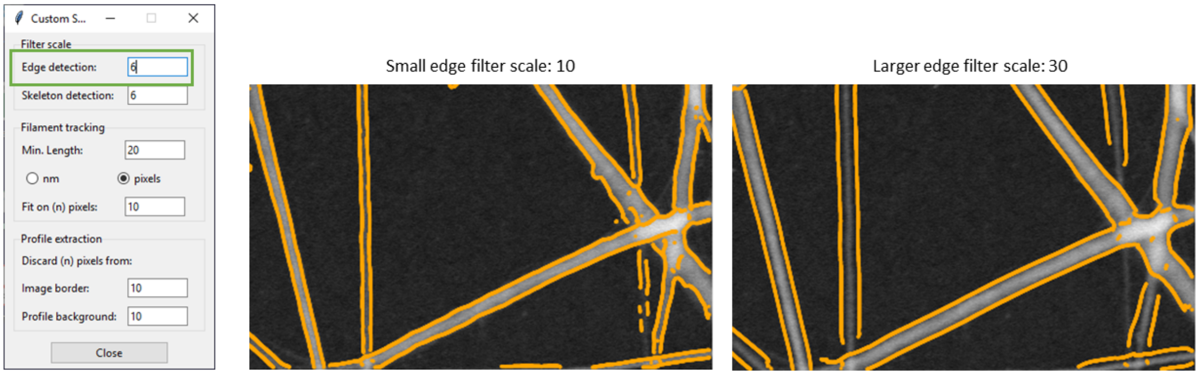


Figure 5: **Kernel filter scale in in edge detection.** The scale of the filter can be modifiied in the `Advanced Settings` module. The larger the scale, the filter would be stronger, and the edge detection will be smoothed and wider.

The main parameter to optimise to ensure a good edge detection is the value of the high threshold (`[Control Panel] -> Edge detection options -> Threshold High`)(Fig. 6). The value of the threshold is taking into account the normalised gradient, thus, it's a fraction of the *maximum* gradient value for that image. Each time the value is modified, you need to click on `Run` to update the results. If the scale of the kernel has been kept constant, the program will run again only the threshold algorithm; if the scale was modified, it will run the entire edge detection algorithm, including the updated value for threshold. **Tip:**

If there are a lot of intensity saturated structures (high gradient), you can check the `Enable upper limit` box, to discard all the edges *above* that value.

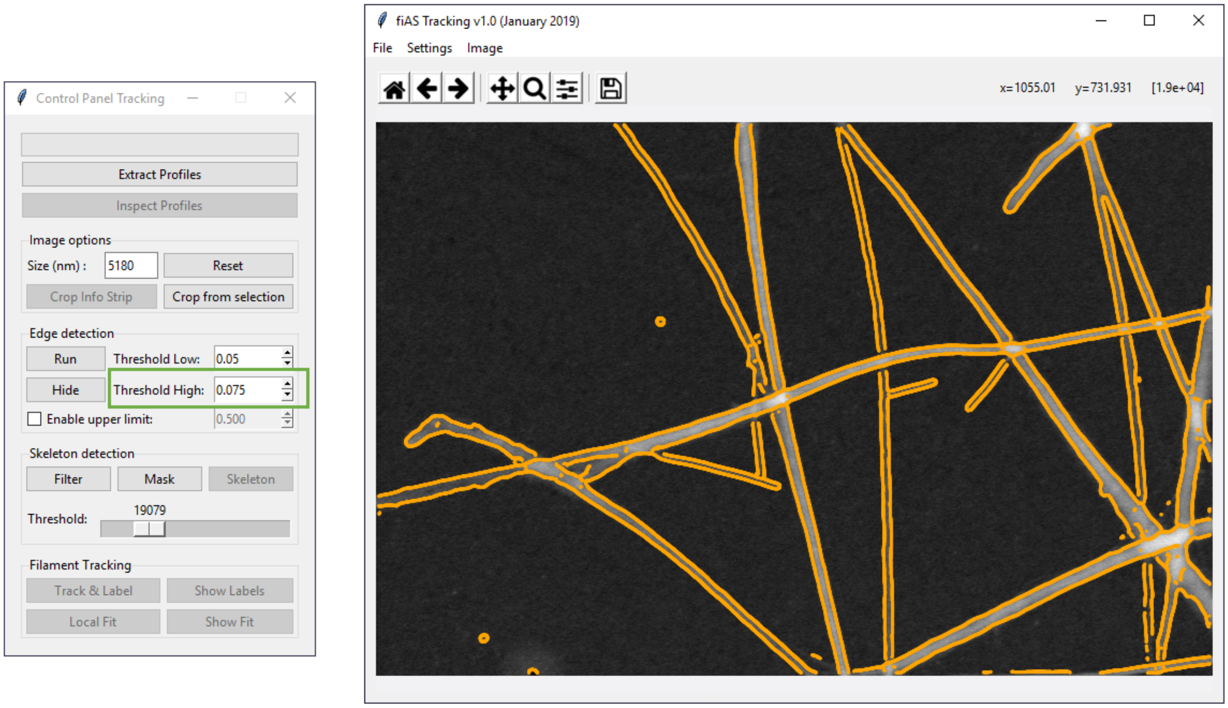


Figure 6: **Optimised tracking of filament edges**. By optimising both the filter scale (in advanced settings) and the value of the `Threshold High` (highlighted with the green rectangle), the tracking of the edges can be improved. In the above image, the `Filter scale` was set to `10`.

## 2.3 Skeleton Detection

**Quick instructions: Skeleton Detection**

```
[Control Panel] [Skeleton detection] -> Filter
[Control Panel] [Skeleton detection] -> Mask
[Control Panel] [Skeleton detection]  -> Adjust Theshold slider
[Control Panel] [Skeleton detection] -> Skeleton
```

**Tip:** Although the skeleton detection is independent from the edge tracking, it is recommended to run it afterwards, since it can use some of the results from the edge tracking process as an input to automatise some tasks.

Just like the edge detection, the skeleton tracking relies on a filtering kernel as a first step in the algorithm. In this case, the image is only smoothed, therefore only a gaussian kernel is used. After smoothing, the image is binarised based on an intensity threshold, and later this binary mask is thinned until the skeleton is extracted.

The first step in the skeleton tracking algorithm is to smooth the image. This is done by using a gaussian filter as a kernel, whose width or scale can be modified in the advanced settings (`[Menu] -> Settings -> Advanced Settings`). The larger the scale, the stronger the smoothing effect (Fig. 7). Most of the images

will require just a minimum of smoothing, as the requirements of the mass mapping technique result in a clean background. However, if there are a lot of details in the filaments, it could be better to go to stronger smoothing values, to reduce tracking of these details.

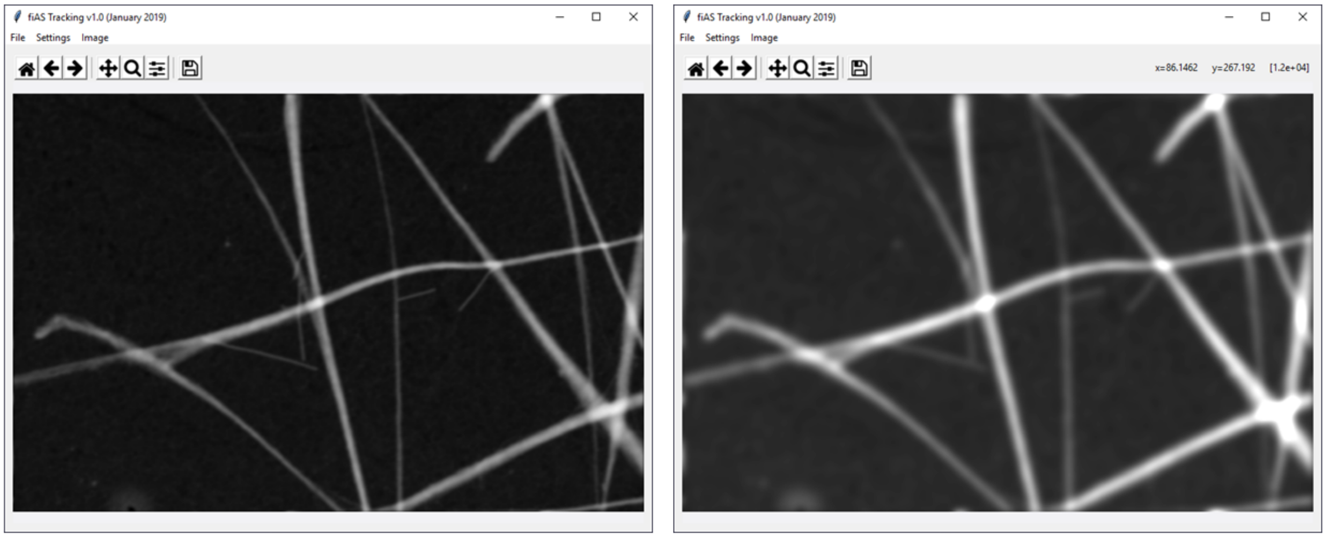


Figure 7: **Image filtering for skeleton detection.** The `Filter scale` for the gaussian kernel can be modified to have a weak smoothing (left panel, `scale = 6`) or stronger smoothing (right panel, `scale = 12`).

Once the image has been filtered, it can be converted to a binary mask by selecting an intensity threshold. If the edge detection has already been performed, the program will automatically choose the value of the threshold based on the average intensity value found at the detected edges. The binary mask is shown as an overlay on the image (Fig. 8). The user can use the `Slider` to adjust the threshold value. Note that it is not necessary to capture the whole width of the filament, as the mask will be thinned to an skeleton.

Finally, the skeleton can be obtained with the (`[Control Panel] -> Skeleton detection options -> Skeleton`)(Fig. 9). Note that the skeleton can have extra branches in some parts, and skeletonization can run into troubles when a junction is encountered. Although it is possible to discard bad points at any point in the tracking or analysis process, it is recommended to do so at this point. By choosing only the filaments or areas that will be analysed, the profile extraction will be faster. To select points, use the `ROI manager` found under the `[Menu] -> Image` options.

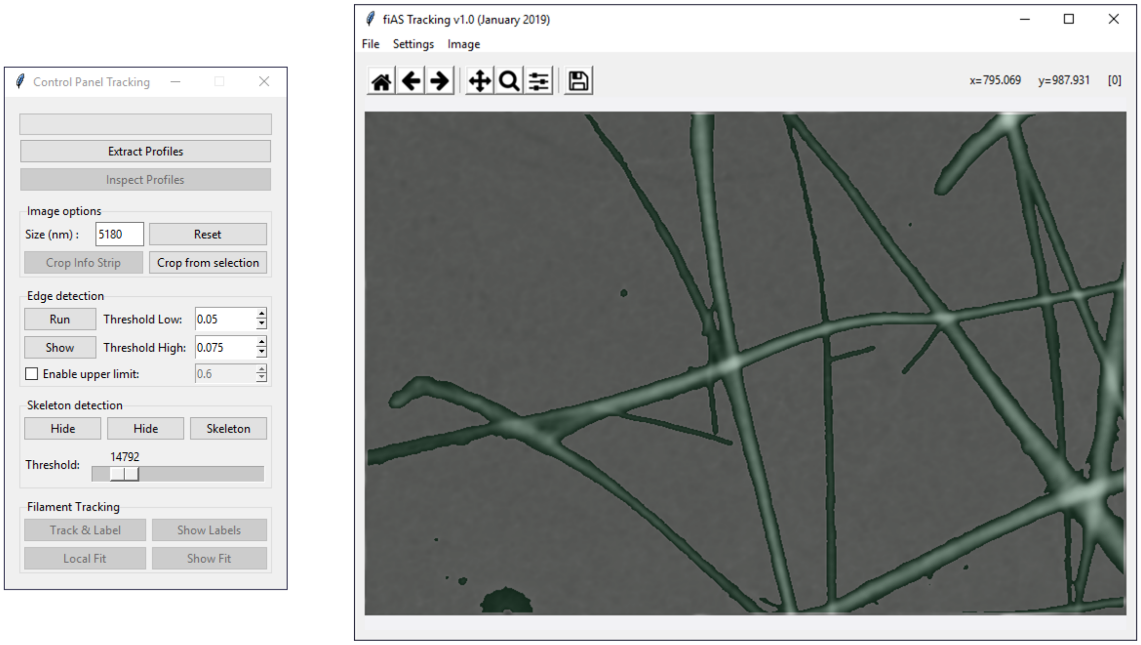


Figure 8: **Binary mask for skeleton detection.** After filtering, the image can be binarised using the `Mask` option and adjusting properly the `Threshold Slider`. In the image shown, the threshold value has been adjusted to optimise the binary mask.

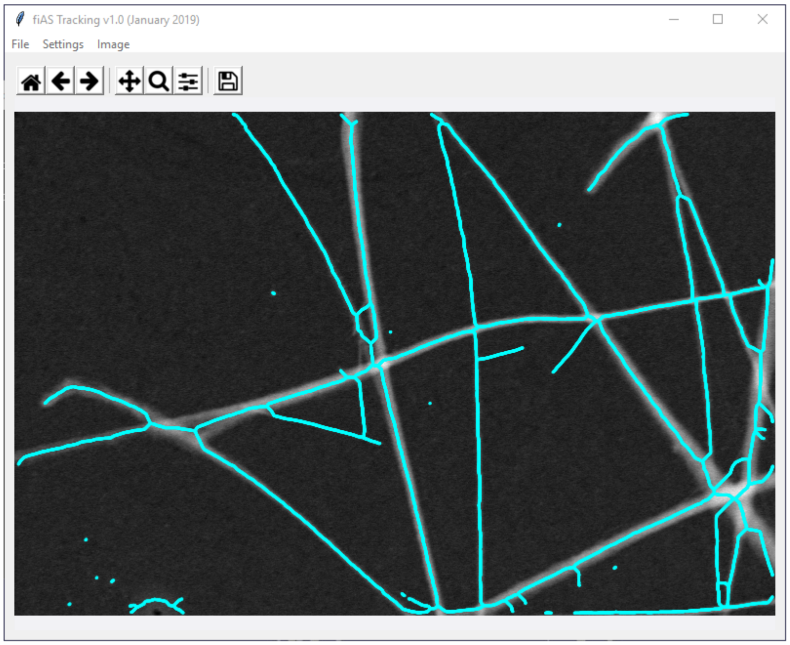


Figure 9: **Skeleton detection.** The binary mask is thinned to obtain the skeleton of the filaments.

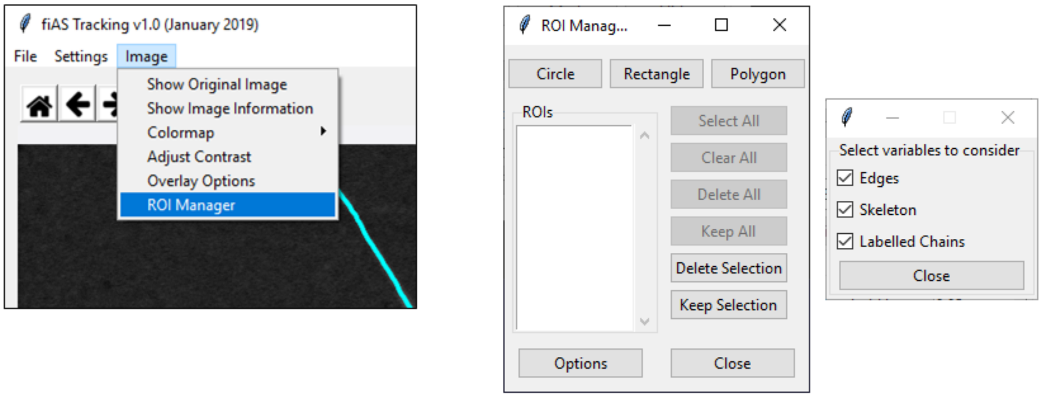


Figure 10: **ROI manager in the tracking module**. Points can be discarded in any step after detection by using the `ROI manager`. It is possible to select which feature is taken into account by using the `Options` of the ROI manager window.

The ROI manager can be used at any point after detection. By drawing circles or rectangles (Fig. 11), it is possible to select or discard the desired points or regions. Once a ROI has been drawn, it is added to the list of the manager with a unique identifier. From this list is then possible to `Delete All` the points inside the ROIs in the list, or the opposite option of `Keep All`, which eliminates all the points outside of the ROIs in the list. If only a subselection of ROIs is wanted to delete or keep the points, the user can select the desired ROIs in the list, and then use the options `Delete Selection` or `Keep Selection`. The list can be cleared by using the `Clear All` option, which deletes the ROIs in the list without modifying the tracked points. The user can select which features will be used in the point selection, using the `Options` button. It is possible to choose between `Skeleton`, `Edges`, and `Labelled Chains`. Any combination is possible. Note that at this stage, all the features are independent from each other, as the edges have not been assigned to their corresponding skeleton point. The program will skip any feature that has not been detected.

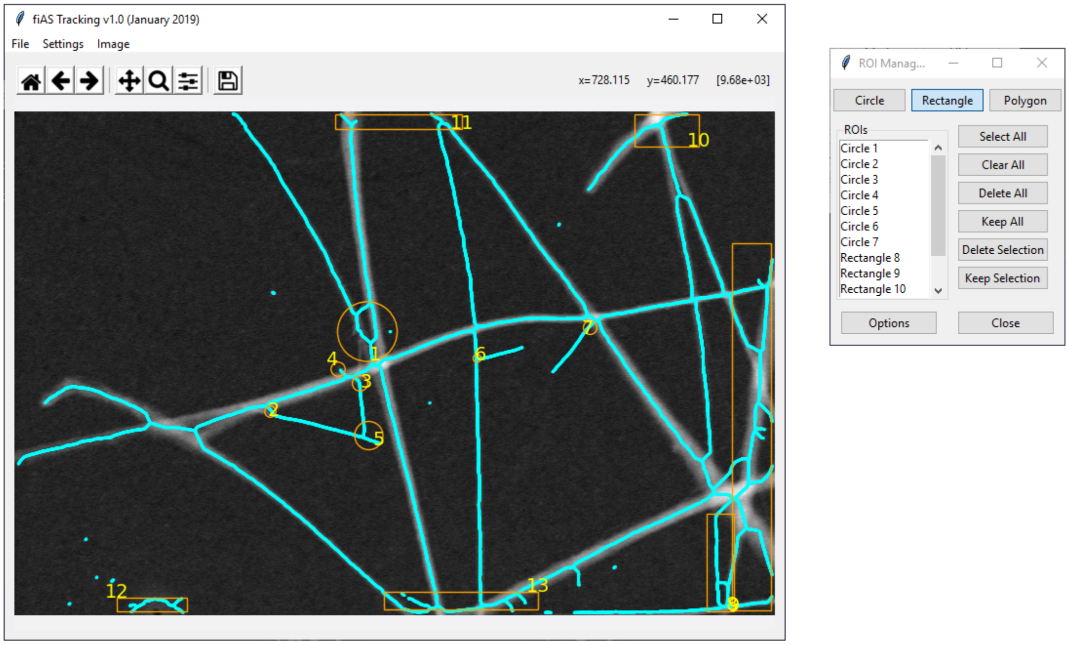


Figure 11: **Discarding points with the ROI manager on the tracking module**.

## 2.4 Filament Tracking and Fitting

The next step in the tracking algorithm chains together the skeleton points, labeling with a unique number that identifies the points as part of the same chain or filament. One of the embedded actions involved in this step consists in discarding chains with a length below a certain threshold. The threshold value can be modified in the `Advanced Settings` panel, on the `Min.Length` option, and it can be given in `nm` or `pixels` (**NOTE**: the `pixels` option hasn't been fully tested; if the user wants to choose it, it is advised to run a verification test). The output of the tracking method is shown on the main display by using the same colour for the point belonging to the same filament (Fig. 12).

**Quick instructions: Filament Tracking and Fitting**

```
[Control Panel] [Filament Tracking] -> Track and label
[Control Panel] [Filament Tracking] -> Local Fit
```

Finally, there is a local linear fit performed around each point to determine the main axis of the filament, and to be able to trace the cross-section to extract the intensity profile across the filament. The extent of the fit, that is, the number of pixels that are taken into account for the local fit, can be tuned in the `Advanced Settings` panel, under the option `Fit on (n) pixels`. Note that the point for which the linear fit is performed, will be placed at the middle of this range, thus, there will be `n/2` pixels before, and `n/2` pixels after the selected point. The linear fit is a time-consuming step that can take from a few seconds to a minute or two. The results of the fit will be shown on the main display once the process is finished (Fig. 12).

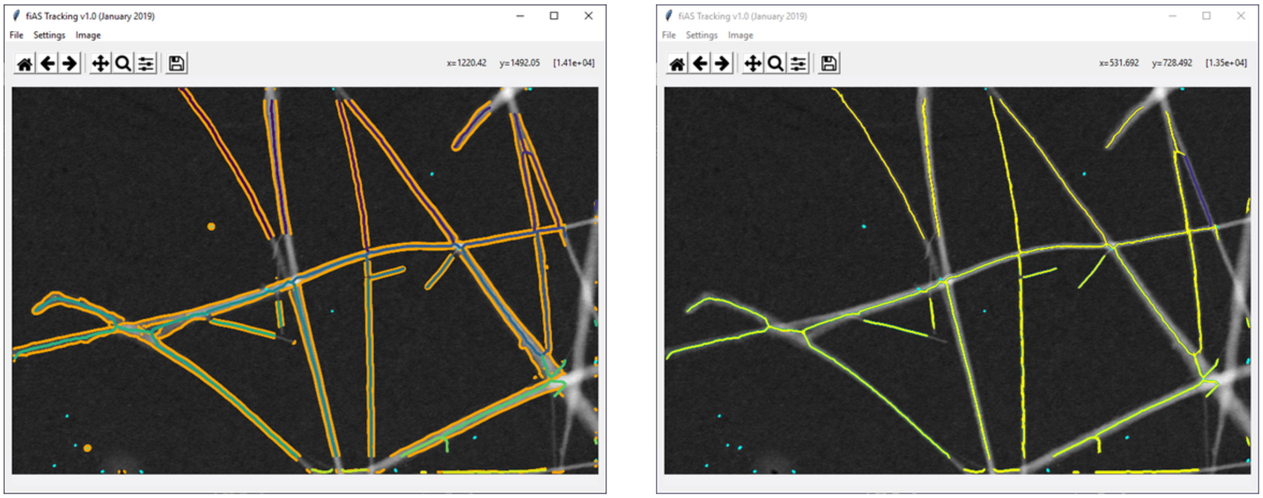


Figure 12: **Filament tracking and fitting**. The chained points will be shown in different colours, with each colour corresponding to the filament number (left pannel). After tracking, it is possible to perform a linear fit. The quality of the fit can be evaluated by inspecting the reconstituted lines around each point (right pannel). Note that the length of the line corresponds to the length of the fit that was used.

## 2.5 Extract Profiles

The last step to obtain the profile data is to extract the profiles. This is a very time-consuming step, that can take from a few minutes to several hours. It is therefore recommended to let it run on the background, but trying not to do many heavy memory-consuming tasks. Once the user clicks on the button `Extract Profiles` i nthe `[Control Panel]`, all the other buttons will be disabled as well as the main display window,

although it is still posible to minimise the windows. The progress will be shown in the `[Command Prompt]`, where the profile number, and the filament number that are currently being extracted, as well as the total detected, are shown (Fig. 13). **Tip:** To stop the process, press `<Ctrl-c>` while the command prompt is active, this will abort and force the program to close (All progress will be lost!).

When the program finishes extracting profiles, there will be a pop-up window indicating the total number of filaments extracted. Close this window and go back to the main window. It is possible now to save the data.

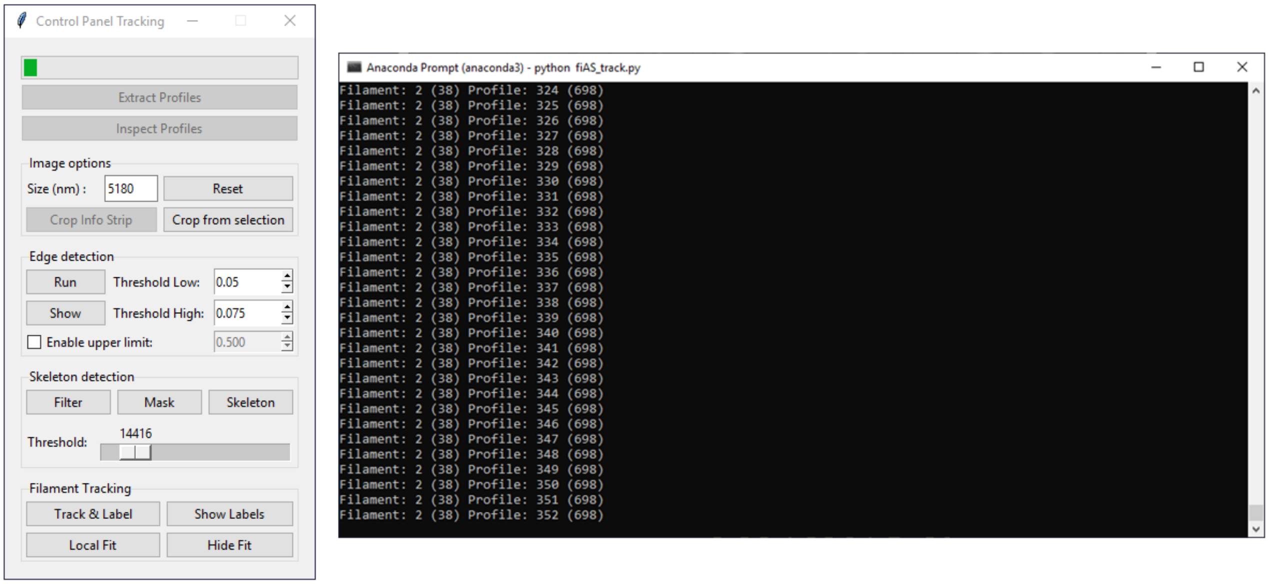


Figure 13: **Extracting Profiles in the tracking module.** When performing this step, the control panel will be inactivated, and the command prompt will show the progress.

## 2.6 Saving Data

The results have to be manually saved, by going to the menu on the main window `[File] -> Save As`. A window will appear asking the user to select the directory and the name of the file where the results will be saved. Data is saved as a `.npz` file. The file contains the cropped image used for tracking (`mat_img`), the calibration factor to convert pixels to nm (`cal_factor`), the intensity and position profile for each cross-setion of the filament skeleton (`int_profile, pos_profile`), the filament or chain number (`fiber_number`), the slope from the local linear fit (`fiber_slope`), the coordinates for the skeleton points (`fiber_xc, fiber_yc`) and the coordinates for the detected edges (`fiber_x1, fiber_x2, fiber_y1, fiber_y2`).

It is highly recommended to also save the settings used for the filament tracking. This can be done by going to the menu on the main window `[Settings] -> Save Settings`. A window will appear asking the user to select the directory and the name of the file where the settings will be saved. The file is a `.txt` file containing all the parameters used.

At this point, the program can be closed. Note that it is best to fully close the program to erase any data that can interfere with the tracking of a second image. Also it is advised to wait a few seconds before opening another instance of the program, to allow for the memory to be cleared properly.

# 3 Analysis Module

The results from the Tracking module can be imported to the Analysis module to compute the integrated intensity, filament width, and if the intensity-mass relation is known, also the mass per unit length.

As soon as the analysis module is launched, the corresponding window will appear (Fig. 14). Just as with the tracking window, most of the options are disabled at this stage, as they cannot be operated. The first step is therefore to load the results. Go to `[File] -> Load`. A window will appear showing the possible data to import (Fig. 14, small window). The user can decide what data to import. However, please note that the coordinates as well as the profile data are required to compute the integrated density and do mass mapping.

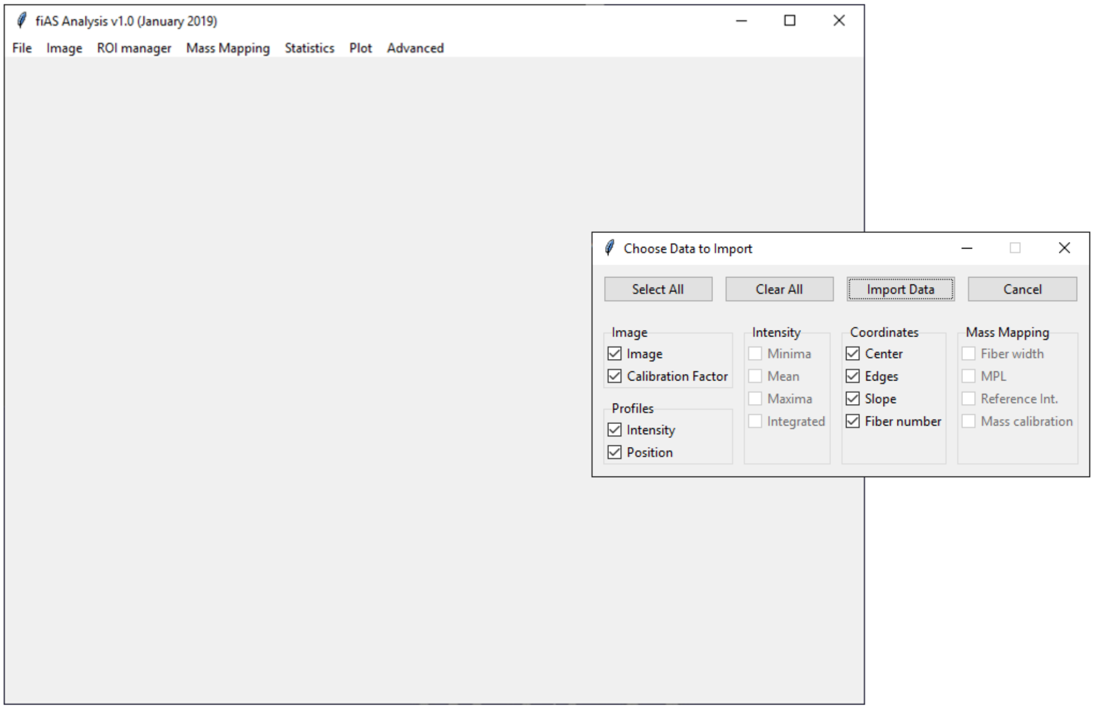


Figure 14: **Analysis Module.** When launching the program on the command prompt, the main window where the analysis operations can be done will appear (big window on the figure). The first step is loading the data using the menu `[File] -> Load`, the user can choose which data to import using the Import window that pops-up upon loading (small window on the figure.)

**NOTE: Limited functionality in analysis**

The current version of the GUI program has a limited functionality in the analysis actions that can be taken. The options for `Statistics, Plot, Advanced` are at the moment not enabled. These options are under development and will be added in upcoming releases. The preliminary code used for those options can be shared with the user upon request. Please note that if the user would like to use the preliminary code, it will be necessary to adapt the code to their data, and will need to run it using an interpreter for python or in the command prompt.

## 3.1 Cleaning the Data

Once the data is imported, it will appear on the main display (Fig. 15). Note that some of the detected points have not been correctly tracked. Errors in the final tracking can occur due to a poor local linear fit that cannot retrieve the right cross-section, erroneous inner edges detected, or the absence of edge points in the cross-section of the filament skeleton. The skeleton of the filament is recalculed once the corresponding edges have been detected, by placing the skeleton in the midpoint of the edges, causing some of the skeleton points to be displaced from the main filament axis.

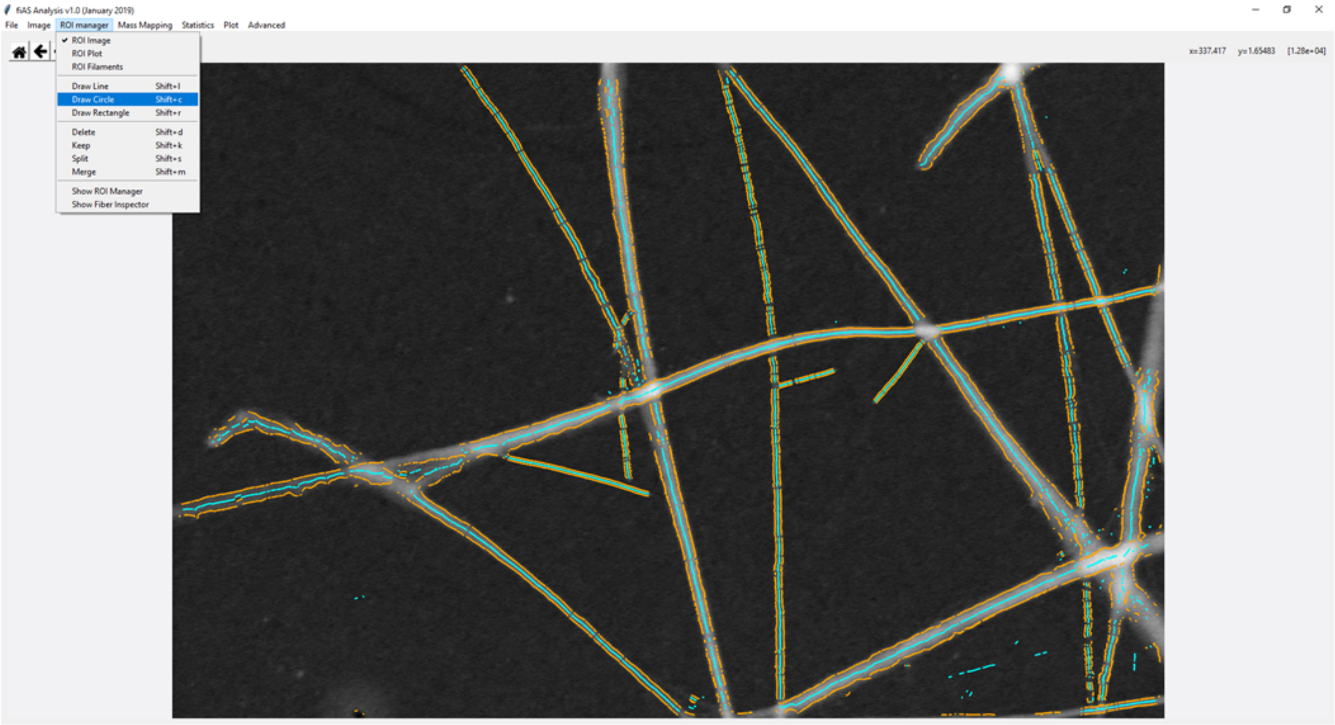


Figure 15: **Imported data and ROI menu options**.

The user can use the `ROI manager` menu to discard all the points that are not properly detected (Fig. 15). At this moment, only the options for `ROI Image, Draw Circle, Draw Rectangle, Delete, Keep` are available. It is recommended to use the keyboard shortcuts provided to save time. The points that are clearly mistracked must be deleted, but also those points where there's no clear background on both sides of the filament. Likewise, caution must be paid to the intensity of the filament, to avoid taking into account any regions with intensity saturation, which will result in incorrect quantification. It is recommended to save the clean data using the menu `[File] -> Save As`, this option will save the data in a similar way to the saving option in the tracking module. Note that the data deletion is based on the points from the filament edge. If one requires to make the deletion based on the skeleton points, it is necessary to change the display mode to show only the skeleton points. To do so, go to `[Image] -> Show Overlay Options`, and change the selection to only show the skeleton points. **Tip:** Whenever the edges are shown, data selection/deletion will be based on the edges enclosed by the ROIs.

## 3.2 Setting the reference for mass mapping

To continue with the mass mapping computation, the next step is to set the reference in the image. Note that if the relation intensity-mass is known or if only the integrated intensity is required, it is possible to skip this step. To set the reference go to `[Mass Mapping] - Set Reference` (Fig. 16). The display will change

to show the unknown filaments in blue, and the references in red. Moreover, the option to `Draw Circle` will be automatically updated, allowing the user to enclose the known references in circular ROIs (inset in Fig. 16).

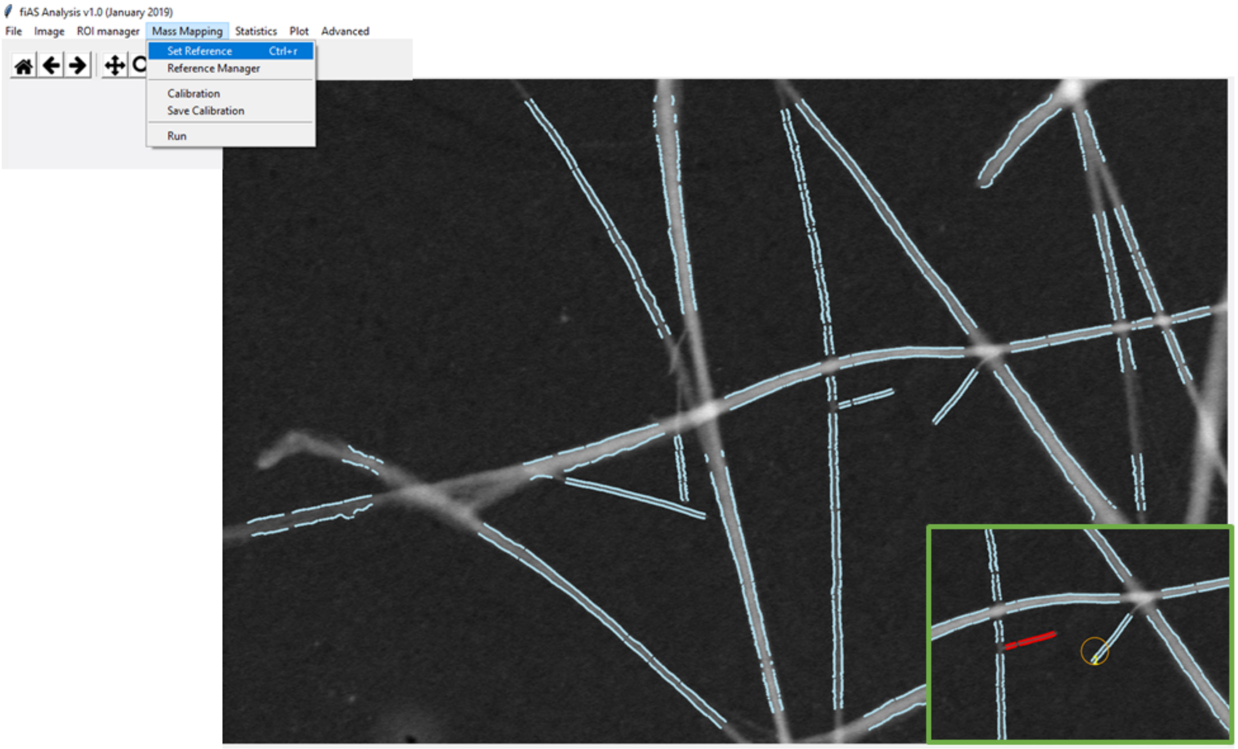


Figure 16: **Set reference for mass calibration.** If the reference for mass calibration is present in the image, it is possible to set those filaments as reference by using the `Mass Mapping->Set reference` option. The display changes to show in blue the filaments that are unkown, and in red those filaments that have been set as reference (inset). The selection of references is made by drawing circular ROIs containing at least one point from the desired filament.

## 3.3 Intensity-Mass calibration

Once all the filaments that can be used as references have been selected, it is possible to perform the intensity-mass calibration. Go to `[Mass Mapping] -> Calibration`. The window with the options for mass calibration will appear (Fig. 17). There are a couple of options predefined, including the Tobacco Mosaic Vorus (TMV) and the Fd rods, but any reference can be used by modifying the parameters of mass per length and width accordingly. At the moment, it is possible to do the intensity-mass calibration just by computing the histogram of the integrated density.

When choosing the histogram option, the program will proceed to compute the integrated density for all the filaments. Then the histogram will be computed for only those filaments that have been selected as reference. Once this is done, a window showing the calculated histogram will appear (Fig. 18). The parameters to compute the histogram are by default based on the data range, with a number of bins set to `10`. However, the parameters can be easily modified by the user in this window. Note that each time that a parameter has been modified, it is necessary to click on `Update histogram` for the changes to take effect. Once the distribution has been properly computed, the next step is to perform a gaussian fit, which will be used to obtain the required intensity-mass calibration. Click on `Fit Data` to run the gaussian fit, a window will appear showing the results of the fit, and the fitted curve will be shown in blue on top of the

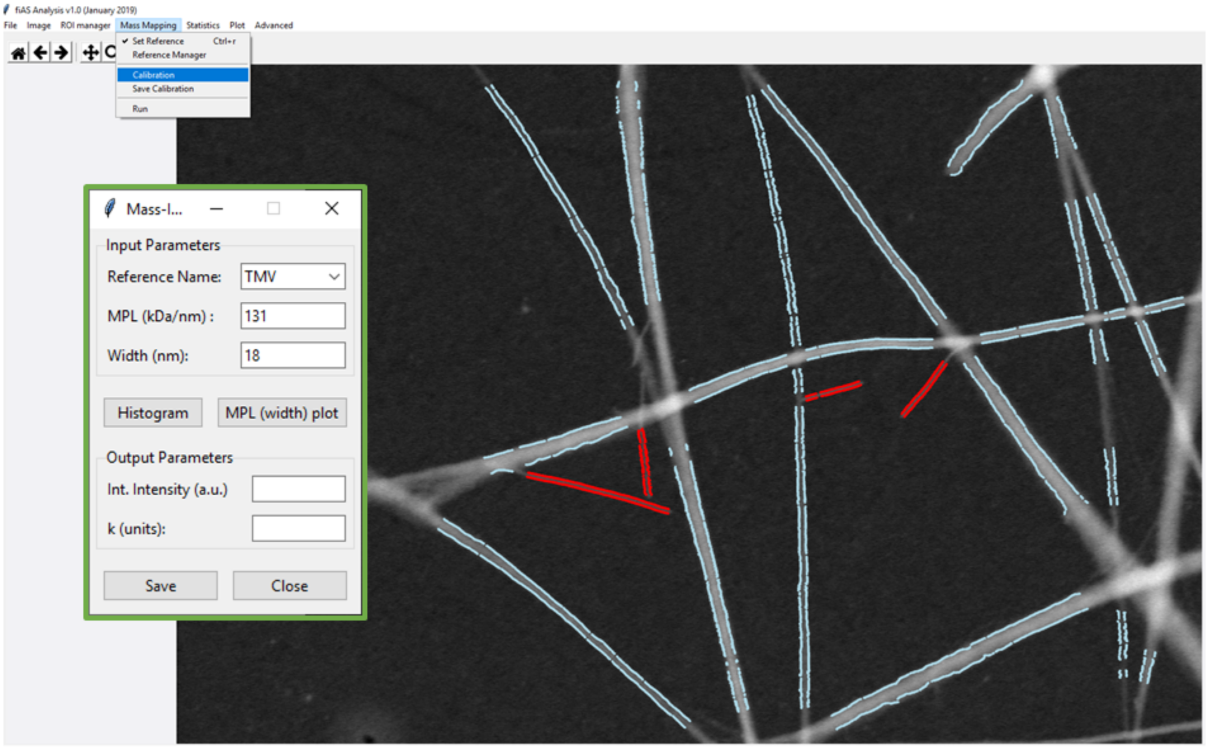


Figure 17: **Reference options for mass calibration.** The calibration options (inset in green) contain the mass per length and width of the reference. This window should remain open until the calibration has been completed.

histogram. If the fit is satisfactory, click on `Save` before closing this window. By saving the results, they will be registered in the memory of the program. The user can check that they have been properly registered by looking into the window showing the mass calibration parameters, where the `Output Parameters` panel should be filled with the results of the gaussian fit. save the results once more by clicking `Save` on the mass calibration window (with the parameters), and close. Now, all the parameters required for the mass computation should be correctly registered in the program memory, and is possible to continue with the mass per length computation.

## 3.4 Mass per length computation

Once the data is ready for the mass per length computation, go to `[Mass Mapping] -> Run`. A window showing the options for computing the mass per length of the filaments will appear. In this window, it is possible to manually input the calibration constants `k, c`, if they're known, or they can be set to `1` if uncalibrated data is preferred. The `Intensity` and `Mass` panels show the information that can be saved. The raw values of mass refer to the uncorrected mass, that is, without subtracting the contribution from the background. The user can also choose whether to keep the profiles after running the mass computation. The default option is set to `No` as they are not longer needed and occupy a significant portion of the memory. The data will be automatically exported at the end of the computation, and also the window will automatically close once the computation is finished. This is a very light operation, that usually is quite immediate. requiring less than one second to be completed.

The data saved contains the mass mapping results. Data is saved as a `.npz` file, containing the parameters chosen to be saved plus the filament number, width, and coordinates of the skeleton and the edges.

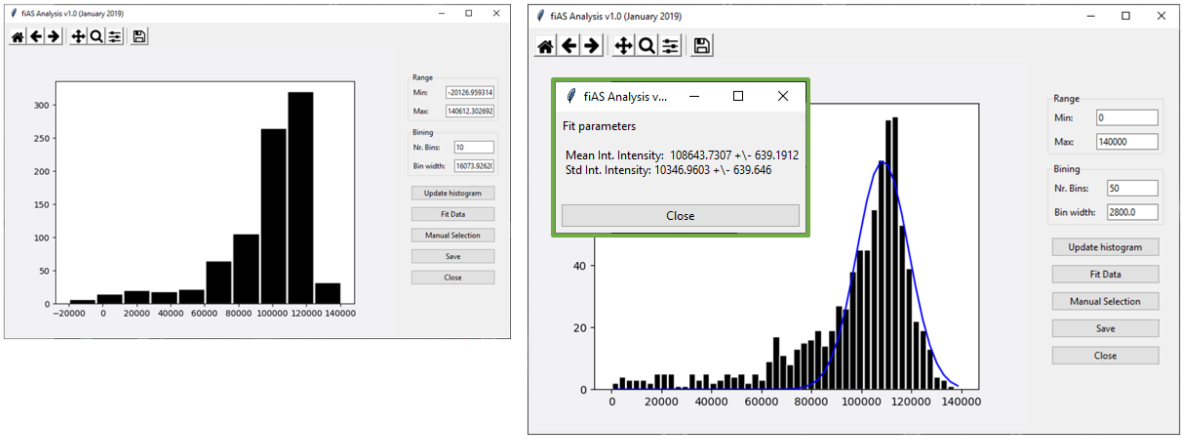


Figure 18: **Mass-intensity calibration based on integrated density distribution.** By default the program computes the histogram using the data range and 10 bins (left), but these values can be optimised (right) to improve the gaussian fit. Once the user is happy with the histogram, it can fit the data and the results will appear in a new window (green inset).

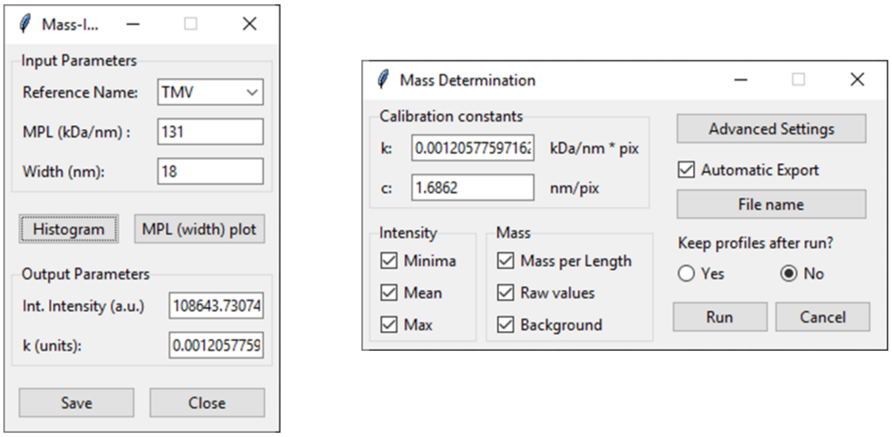


Figure 19: **Mass mapping computation.** The mass per length computation can be done on `[Mass Mapping]-> Run`. The window that appears (shown on the right) allows the user to manually input the calibration constants or, if they are known to the program, it fills them in automatically. The user can select which parameters are saved, and whether the profiles are kept in memory after the mass computation is done. The window dissappears when mass computation is done.

# 4 Troubleshooting

For troubleshooting advice please refer to the magenta boxes in this guide showing known issues and possible solutions. If the issue you encounter is not listed here, please report it on the github repository or send an email to c.e.martineztorres(at)tudelft.nl , on the subject line write `fiAS:` at the beginning, followed by your query/report. Note that the issues reported to Github will be addressed quicker.
