# Supplementary material for: Revealing the assembly of filamentous proteins with scanning transmission electron microscopy
Source: PLoS One. 2019 Dec 20;14(12):e0226277. doi: 10.1371/journal.pone.0226277 (PMC6924676; doi:10.1371/journal.pone.0226277)
Supplement: Attachment — Submitted filename: Review of PLoS manuscript_08-08-19.pdf [file pone.0226277.s006.pdf]

Review of PLoS manuscript, PONE-D-19-20842, “Revealing the assembly of filamentous proteins with scanning transmission electron microscopy”

This manuscript describes a very useful technique for automating mass-per-length measurements of protein fibrils and filaments based on STEM annular dark-field imaging. Particularly interesting is the calculation of packing density based on information about the number of monomers per cross sectional area. Importantly, the authors make their data and computer code available through the GitHub repository, so that other users can potentially test the methods on their own data. However, the extent of data and software annotation is not clear, and the practicality of adoption by other users will depend on the ease of usage. In this regard, it would be helpful if the authors included examples of data annotation and code annotation in Supplemental Information in order to encourage other researchers to apply their software. After all, this is surely be the main value of the paper.

Specific Points:

1. Specimen preparation is only mentioned briefly, yet the choice of air drying rather than rapid freezing and cryotransfer is likely to be important for more many types of fibrils or filaments. For example, microtubules composed of hollow tubular arrangements of protofilaments are known to collapse on air drying. The collapse would be evident in AFM images of the same structures, and wrong conclusions could easily be drawn in such cases.

2. Some proteins such as intermediate filaments, e.g., neurofilaments contain long disordered sidearms. It is not clear how such structures would be accommodated within the present framework. In fact, the existence of sidearms can be inferred from the mass-per-length if the number of subunits per cross section is known.

3. In Eq. 1, the quantity $N_{TC}$ is defined as the number of tropocollagen molecules. It would be less confusing to define $N_{TC}$ as the number of tropocollagen molecules per cross section of the filament.

4. In general, the list of references is reasonable. However, the authors should reference the work of V. Krzyzanek, S.A. Mueller, A. Engel, and R. Reichelt, Journal of Structural Biology, Volume 165, pp. 78-87 (2009). “MASDET – A fast and user-friendly multiplatform software for mass determination by dark-field electron microscopy”.
