# Supplementary material for: Revealing the assembly of filamentous proteins with scanning transmission electron microscopy
Source: PLoS One. 2019 Dec 20;14(12):e0226277. doi: 10.1371/journal.pone.0226277 (PMC6924676; doi:10.1371/journal.pone.0226277)
Supplement: Attachment — Submitted filename: Itemized response letter.pdf [file pone.0226277.s007.pdf]

Itemized response
Revealing the assembly of filamentous proteins with scanning transmission electron microscopy

REVIEWER 1

This manuscript describes a very useful technique for automating mass-per-length measurements of protein fibrils and filaments based on STEM annular dark-field imaging. Particularly interesting is the calculation of packing density based on information about the number of monomers per cross sectional area. Importantly, the authors make their data and computer code available through the GitHub repository, so that other users can potentially test the methods on their own data.

We thank the reviewer for the positive appraisal of our work and the constructive comments, which have helped us to strengthen the manuscript.

However, the extent of data and software annotation is not clear, and the practicality of adoption by other users will depend on the ease of usage. In this regard, it would be helpful if the authors included examples of data annotation and code annotation in Supplemental Information in order to encourage other researchers to apply their software. After all, this is surely be the main value of the paper.

We agree that it is important to encourage the application of our software by other researchers by providing software and data annotation, especially given the broad range of possible applications of our software in fields ranging from biophysics to materials science and chemistry.

<u>Action:</u> *We have written a 'quick user guide' to explain the basic functionality of the software and provide guidance on troubleshooting. We furthermore provide a test image for the user to try and get familiarised with the software. We include this quick guide as supplementary information and we have also added it to the Github repository hosting the code. The test image is now also available to download together with the code.*

1. **Specimen preparation is only mentioned briefly, yet the choice of air drying rather than rapid freezing and cryotransfer is likely to be important for more many types of fibrils or filaments. For example, microtubules composed of hollow tubular arrangements of protofilaments are known to collapse on air drying. The collapse would be evident in such cases.**

We had indeed only briefly discussed this important point in the original manuscript version. Since the main purpose of our new method is to determine the mass of the filaments, we chose the simplest method for sample preparation, which does not require any specialised equipment, such as rapid freezing and cryotransfer, increasing the applicability of the mass mapping technique. Our width measurements show that actin filaments and microtubules appear wider on the EM grid than they are in solution, which could be due to collapse on air drying. Therefore, if the main application is to determine precisely the width of the filaments as close as possible to their native state, one should take further measures in sample preparation, such as physical or chemical fixation.

<u>Action:</u> *To emphasize the importance of specimen preparation, we have added the following text (shown in a red font) in our manuscript, lines 144-151:*

> *On the other hand, the sample preparation can also change the width of the fibrils compared to the hydrated diameter in solution due to surface adsorption and drying. To prevent changes in the fibril width and in extreme cases, possible structure collapse, it is preferred to use physical or chemical*

> *fixation, for example by freeze drying, vitrification [24] or glutaraldehyde fixation. Since we avoided chemical fixation in order not to change the M_L, the actin filaments likely flatten upon surface adsorption. As we were mainly interested in the mass of the filaments, we opted to use a simple method for sample drying, which does not require any specialized equipment.*

2. Some proteins such as intermediate filaments, e.g., neurofilaments contain long disordered sidearms. It is not clear how such structures would be accommodated within the present framework. In fact the existence of sidearms can be inferred from the mass-per-length if the number of subunits per cross section is known.

We thank the reviewer for raising this interesting point. The existence of sidearms or other disordered structures can indeed be inferred from the mass-per-length if the number of subunits per cross section is known. In fact, we have already applied our method to intermediate filaments in Lopez C.G. et al, Soft Matter (2018).

Action: *We have added the following text in our manuscript, lines 323 - 329:*

> *There are many examples of such hierarchical fibril-forming proteins in nature where the hierarchy is essential for the mechanical and biological function. Axially periodic packing for instance ensures toughness and biological recognition in collagen [45,46] and high resilience in fibrin [47]. Although we have focused on the description of ordered structures and molecules, mass mapping can also be used for filaments containing disordered regions, such as intermediate filaments [48] or fibrin [49], where information on the existence of unstructured sidearms can be inferred.*

And the references:

- Lopez CG, Saldanha O, Aufderhorst-Roberts A, Martinez-Torres C, Kuijs M, Koenderink GH, Köster S, Huber K. *Effect of ionic strength on the structure and elongational kinetics of vimentin filaments.* Soft Matter. 2018; 14: 8445-8454.
- Protopopova AD, Litvinov RI, Galanakis DK, Nagaswami C, Barinov NA, Mukhitov AR, Klinov DV, Weisel JW. *Morphometric characterization of fibrinogen's αC regions and their role in fibrin self-assembly and molecular organization.* Nanoscale. 2017; 9: 13707-13716.

3. In Eq.1, the quantity NTC, is defined as the number of tropocollagen molecules. It would be less confusing to define NTC as the number of tropocollagen molecules per cross section of the filament.

Yes, we agree and we thank the reviewer for the suggestion.

Action: *We have modified the definition of NTC in the text:*

Lines 225-229:

> *where N_{TC} is the number of tropocollagen molecules per cross section of the filament, and α is the power law describing the fibril packing density that can adopt values in the range of $1 \leq \alpha \leq 2$, with α = 1 (dashed gray line in Fig 4B) corresponding to a quasi-bidimensional or flat structure, and α = 2 (dashed black line in Fig 4B) when the molecules uniformly occupy the interior of a cylindrical fibril.*

Lines 277-279:

> *We take for the positions of the overlap region, s_{over}, each of the local maxima observed, and the number of tropocollagens per cross section in the overlap, N_{over}, as the value of $M_L\ (s_{over})/\ m_o$.*

Caption of Fig. 5.

> *(E) Ratio between the number of tropocollagen molecules per cross section in the overlap (N_{over}) and gap (N_{gap}) regions of the profile shown in (D).*

4. **In general, the list of references is reasonable. However, the authors should reference the work of V. Krxyzanek, S.A. Mueller, A. Engel, and R. Reichelt, Journal of Structural Biology, Volume 165, pp. 78-87 (2009) "MASSDET – A fast and user-friendly multiplatform software for mass determination by dark-field electron microscopy".**

We apologise for missing this reference.

Action: *It has been added to the manuscript on line 62, as reference 14.*

REVIEWER 2

The work of Martinez-Torres et al focuses on the use of STEM to probe the molecular mass and packing of proteins inside filaments. Even though the method has been developed two decades ago, the bibliography is rather thin, most probably because few groups were able to handle the method. In the past, the method was applied on single proteins mostly whereas Martinez-Torres et al. apply it on thicker specimens.

We thank the reviewer for the constructive comments on our manuscript. As detailed below, we have clarified the imaging settings and role of sample thickness.

1. **The bibliography covers the field, even though it would have been appreciated if more references were added, especially about other methods to characterize the structure of proteins such as AFM-IR or other methods based on interaction with light (lasers or synchrotron radiations).**

We had indeed focused the bibliography on studies of protein filament mass rather than structure. However, we agree with the reviewer that STEM provides structural information as well, and it is therefore interesting to compare it with methods using light to characterize protein structure. One important advantage of STEM that we demonstrate in our work is the possibility to map variations in mass between protein fibrils and also within a given fibril. To our knowledge the only other technique that can provide structural information at the single fibre and nanometer level is AFM combined with IR or Raman. This method was recently applied to amyloid fibrils. X-ray scattering measurements using synchrotron radiation can also provide information on protein filament structure and packing density, but these measurements inherently provide ensemble-averaged information.

Action: *We added the following references in the main text and discuss the differences between the types of information that can be obtained from these various methods*:

Fourier transform infrared nanospectroscopy

- Amenabar I, Poly S, Nuansing W, Hubrich EH, Govyadinov AA, Huth F, Krutokhvostov R, Zhang L, Knez M, Heberle J, Bittner AM, Hillenbrand R, *Structural analysis and mapping of individual protein complexes by infrared nanospectroscopy.* Nat Commun. 2013; 4:2890.

For Tip-Enhanced Raman Spectroscopy (TERS):

- Kurouski D, Deckert-Gaudig T, Deckert V, Ledney IK. *Structure and composition of insulin fibril surfaces probed by TERS.* J Am Chem Soc. 2012; 134: 13323-13329.
- vandenAkker CC, Deckert-Gaudig T, Schleeger M, Velikov KP, Deckert V, Bonn M, Koenderink GH. *Nanoscale heterogeneity of the molecular structure of individual hIAPP amyloid fibrils revealed with tip-enhanced Raman spectroscopy.* Small. 2015; 11:4131-9.

- Deckert-Gaudig T, Kurouski D, Hedegaard MA, Singh P, Ledney IK, Deckert V. *Spatially resolved spectroscopic differentiation of hydrophilic and hydrophobic domains on individual amyloid fibrils.* Sci Rep. 2016; 6: 33575.

For synchrotron radiation:

- Misof K, Rapp G, Fratzl P. *A new molecular model for collagen elasticity based on synchrotron X-ray scattering evidence.* Biophys J. 1997; 72: 1376-81.

The following text has been added on the manuscript, lines 301-310:

> *Although different methods can be used for measuring the molecular packing structure of proteins, e.g., small angle scattering using synchrotron radiation and optical turbidity ~~techniques~~ measurements, they rely on bulk measurements and often involve assumptions about the network or protein structure to support the data analysis [38-40]. The spatial resolution and correlative image-mass analysis of our approach allows us to retrieve, in a label-free manner, information about the filament mass per length, molecular packing density, and about features related to the axial and longitudinal assembly of the protein. This information complements the structural information one can obtain by nanoscale infrared and enhanced Raman spectroscopy measurements, which provide spatially resolved information on the secondary structure of protein fibrils [41-44].*

2. **The work is well presented, showing step by step the validation process, then the deeper and deeper characterisation of filaments. The discussion lacks concerns about how the sample has been prepared. What about sample deformation induced by the beam, sample deformation inside vacuum, etc.?**

Biological samples can be easily deformed, therefore, it is very difficult to discriminate between the deformation induced by the imaging in the electron microscope itself from deformation induced by the sample preparation. For this reason, we had included an experiment where we performed AFM imaging on a collagen sample that had been imaged also with STEM. Our results, as illustrated in Fig. 3B, showed no significant difference, indicating that deformation due to the electron beam and vacuum are insignificant. The sample preparation can also cause fibril deformation. Our width measurements show that sample preparation by air drying gives rise to some flattening in case of actin filaments and microtubules. Therefore, if one is interested in determining precisely the width of the filaments as close as possible to their native state, one should take further measures in sample preparation, such as physical or chemical fixation.

<u>**Action:**</u> *We now discuss more extensively the importance of verifying whether the electron beam or vacuum deform the sample in the manuscript lines 312-319:*

> *Our findings demonstrate that STEM has a wide mass range, from 17 kDa/nm for Fd bacteriophages and actin filaments to 20 MDa/nm for collagen bundles. However, we caution that attention must be paid to the thickness of the specimen [14], which should not exceed ~ 200 nm, and to possible sample deformation induced by the beam or the vacuum. We therefore recommend to validate the fibril thickness with an independent technique such as AFM, which, as we showed, can be performed on the same EM grid as used for STEM imaging.*

3. **Also, the potential of the method on cryo-samples would be very interesting for the reader.**

It would indeed be interesting to extend the method in future to cryo-samples, where the fibrils are physically fixated by vitrification of the aqueous medium.

Action: *We added a remark on this in Line 147 together with the following Reference:*
Elbaum M. *Quantitative cryo-scanning transmission electron microscopy of biological materials.* Adv Mater. 2018; 30: e1706681.

Specific concerns:

4. **The first one is the lack of information about the parameters of the electron beam. The source current, the electron dose, the dwell time, the convergence semi-angle and the inner and outer collection angles are not defined. Without these numbers it is not possible to know the shape of the beam and what kind of electron/information was recorded on the HAADF detector.**

We have added the information on the electron beam parameters in the Materials and methods section of our manuscript, lines 369-372:

Imaging was performed using the High-Angle Annular Dark-Field mode (HAADF) on a Verios 460 electron microscope (FEI, Hillsboro, OR, USA). The beam current was set to 100 pA and pixel dwell time to 3 μs, resulting in an electron dose of 400-1000 e^{-}/nm^{2} with a convergence semi-angle of 2–10 mrad, depending on the specimen.

5. **The second concern is more important. STEM mass measurements using the HAADF detector can be safely performed on thin samples since electrons do not scatter more than once on thin samples. However, when thick samples are studied, HAADF signal is not fully representative of the sample (STEM tomography on thick samples is always performed in BF for the same reason). Furthermore, the convergence of the beam also has to be carefully set to very low values to avoid merging information from outer portions of the sample. Beam broadening is also another issue. Also, the results will be different if the beam was focused at the top or at the bottom of the filament. Based on the scale bars displayed in Fig. 4, the filaments can often measure over 200 nm, which is no longer in the thin sample range. In these conditions, the beam reaching the top of the filament and exiting the filament might have relatively large diameters, collecting blurry information from areas larger than the one where the beam is focused. The measurements made on thin samples might be valid (and the TMV rods used as reference is a good idea), however, for thicker filaments (telo and atelo) the values are probably wrong, likely explaining the difficulty to describe the values of the curves presented in Fig. 4.**

Mass quantification on thick samples is indeed outside the scope of our method. However, in our manuscript all specimens are thinner than 200 nm. Although the width of the collagen filaments can indeed be larger than 200 nm, their thickness is under 100 nm, as measured by AFM (see Fig. S2), likely due to a combined effect of dehydration and sample adsorption. In the case of the collagen fiber bundles, the thickness can locally be at the limit of thickness that can still be measured, which is why we do not characterise the fiber bundles in any quantitative detail.

Action: *We now discuss more extensively the importance of verifying that the sample thickness is below 200 nm in the manuscript lines 312-319 (see reply to Comment 2). We furthermore explicitly point out that we refrain from analyzing collagen fiber bundles because they locally exceed this*

threshold thickness, and we point out that the analysis of thick specimens should take into account multiple scattering as explained in the following reference, which was added:

Krxyzanek V, Mueller SA, Engel A, Reichelt R. *MASSDET – A fast and user-firendly multiplatform software for mass determination by dark-field electron microscopy.* J Struct Biol. 2009; 165: 78-87

6. **The authors are asked to give the microscope settings used, and explain how they take into the various beam effects occurring when studying such thick samples.**

   We now provide the microscope settings (see response to Comment 4) and comment on the role of sample thickness (see response to Comment 5).
